# Supplementary material for: The Potential Role of Cytotoxic Immune Effectors in Induction, Progression and Pathogenesis of Amyotrophic Lateral Sclerosis (ALS)
Source: Cells. 2022 Oct 31;11(21):3431. doi: 10.3390/cells11213431 (PMC9656116; doi:10.3390/cells11213431)
Supplement: Supplementary file 1 [file cells-11-03431-s001.zip › cells-1820423-supplementary.pdf]

Supplementary file

Figure legends

Figure S1: Increased percentages of NK and B cells in PBMCs obtained from ALS patients.

PBMCs of healthy individuals and ALS patients were isolated from peripheral blood as described in Materials and Methods section. PBMCs (2 x 10^5 cells) were analyzed using flow cytometer to determine the percentages of CD14+, CD16+CD56+, CD19+, CD3+CD4+, CD3+CD8+, and CD3+CD56+ subsets within CD45+ immune cells **(a** (n=25)**, b** (n=37)**)**

Figure S2: Increased secretion levels of IFN-γ in PBMCs obtained from ALS patients.

PBMCs of healthy individuals and ALS patients were isolated from peripheral blood as described in Materials and Methods. PBMCs were treated with a combination of IL-2 (1000 U/ml) and anti-PD1 (20 μg/ml), 18-20 hours of treatments, the number of cells secreting IFN-γ were determined as spot counts using ELISpot assay **(a** (n=11), **b** (n=15)**)**

Figure S3: Number of NK cells and IFN-γ secretion in OC-expanded NK cells of ALS patient and healthy individual.

OCs and OC-expanded NK cells were generated as described in the Materials and Methods section. OC-expanded NK cells (2 x 10^5 cells) from healthy individual and ALS patient were analyzed using flow cytometer to determine the percentages of CD16+ cells on days 6, 9, 12, and 27 of culture **(a).** OCs and OC-expanded NK cells were generated as described in the Materials and Methods section. The supernatants were harvested on days 6, 9, 12, 15, 19 and 22 from OC-expanded patient and healthy twin NK cell cultures to determine IFN-γ secretion using single ELISA **(b)**.

Figure S4. Autologous and allogeneic monocytes from ALS patients and those of healthy individuals increase NK cell-mediated cytotoxicity and secretion of IFN-γ.

NK cells and monocytes from ALS patients and healthy individuals were isolated from PBMCs as described in Material and Methods section. NK cells were treated with a combination of IL-2 (1000 U/ml) and anti-CD16 mAbs (3 μg/ml). A crisscross NK cells and monocyte co-cultures were performed with 3 sets of NK cells with allogeneic and autologous monocytes. NK cell- mediated cytotoxicity were measured 18 hours after co-culture using standard 4-hour ^{51}Cr release assay against OSCSCs. The lytic units (LU) 30/10^6 cells were determined using inverse number of NK cells needed to lyse 30% of target cells OSCSCs ×100 (a). NK and monocyte co-cultures were performed as described in Fig. S4a. After 18 hours of co-culture, supernatants were harvested and used in ELISA to measure IFN-γ secretion (b).

Figure S5: Different levels of secreted factors by CD8+ T cells of ALS patients.

CD8+ T cells of healthy individuals and ALS patients were isolated from PBMCs as described in Materials and Methods. CD8+ T cells (1x10^6 cells/ml) were treated with IL-2 (100 U/ml) and anti-PD1 (20 μg/ml) for 18 hours before, the supernatants were harvested to determine the levels of IFN-γ using single ELISA (**a** (n=5)**, b** (n=7)). CD8+ T cells were treated with IL-2 (100 U/ml) and anti-CD3/28 antibody (25 μl/ml) for 18 hours before, the supernatants were harvested to determine levels of secreted factors using multiplex assay **(c, d, e)**.

Figure S6. Increased inflammatory cytokines in peripheral blood-derived serum of ALS patients in comparison to those from healthy individuals.

Sera were obtained from the peripheral blood of healthy individuals and ALS patients, and analyzed for the levels of cytokines, chemokines, and growth factors using a multiplex array kit (a (N=5-17), b (n=7)).

Figure S7: CD8+ T cells' supernatant of ALS patients induced higher cell death and differentiation in OSCSCs.

CD8+ T cells of healthy individuals and ALS patients were isolated from PBMCs as described in Materials and Methods. CD8+T cells ($1x10^6$ cells/ml) were treated with a combination of IL-2 (100 U/ml) and anti-CD3/28 antibody (25 μl/ml) before the supernatants were harvested to determine IFN-γ secretion using single ELISA. Supernatant containing equal amounts of IFN-γ from the healthy individuals and ALS patients' CD8+ T cells were added to OSCSCs for 4 days. On day 4, live and dead tumor cells were manually counted after they were stained with trypan blue (a-b), and the surface expression levels of CD44, and CD54 on OSCSCs were determined using flow cytometry (c). IgG2 isotype was used as control.

Figure S8: Percentages of Treg at different time-points during CD4+ T cells differentiation in healthy individuals and ALS patients.

CD4+ T cells of healthy individuals and ALS patients were isolated from PBMCs as described in Materials and Methods. For Treg differentiation, CD4+ T cells (1×10^6 cell/ml) were cultured with ImmunoCult-XF T cell expansion medium supplemented with Immunocult human CD3/CD28 T cell activator (25 μl/ml). On days 10, 18, and 20, the surface expression levels of Foxp3 on CD4+CD25+ cells was analyzed using flow cytometric analysis. IgG2 isotype was used as control.

Figure S9. Weekly NAC injections in ALS patients did not affect the secretion levels of GMCSF in peripheral blood of ALS patient.

Sera were obtained from the peripheral blood of healthy individuals and ALS patients (before and after NAC injection in patient), and analyzed for the levels of GMCSF using a multiplex array kit (n=8).

Table S1: Patient information

Patient Initials	Age	Gender	Confirmed ALS diagnosis
W.O	57	M	Yes
R.S	62	M	Yes
J.C	60	M	Yes
S.G	54	F	Yes
J.C	62	M	Yes

Figure S1

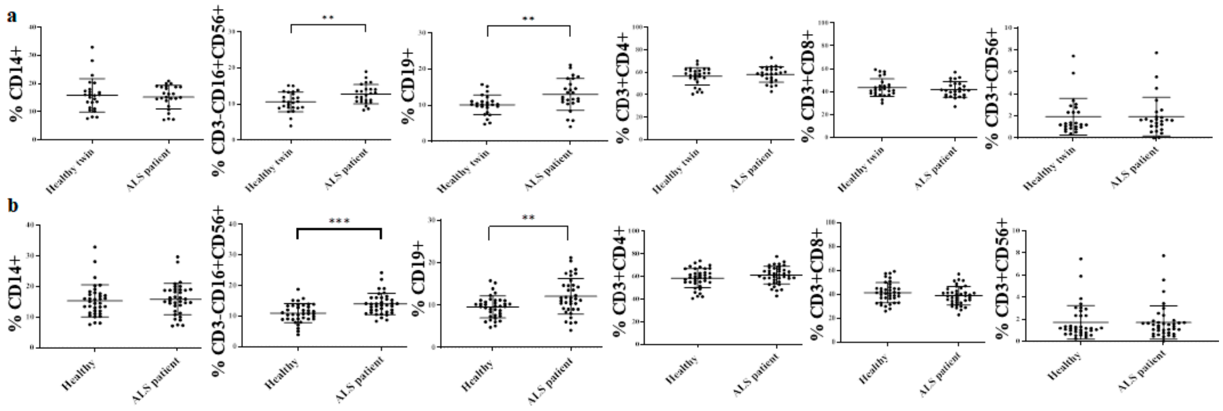

a
% CD14+
% CD3-CD16+CD56+
% CD19+
% CD3+CD4+
% CD3+CD8+
% CD3+CD56+
Healthy twin
ALS patient
**
b
Healthy
***

Figure S2

a

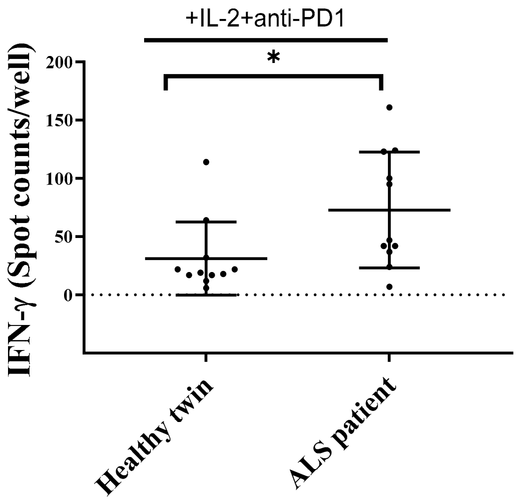

+IL-2+anti-PD1
*
IFN-γ (Spot counts/well)
200
150
100
50
0
Healthy twin
ALS patient

b

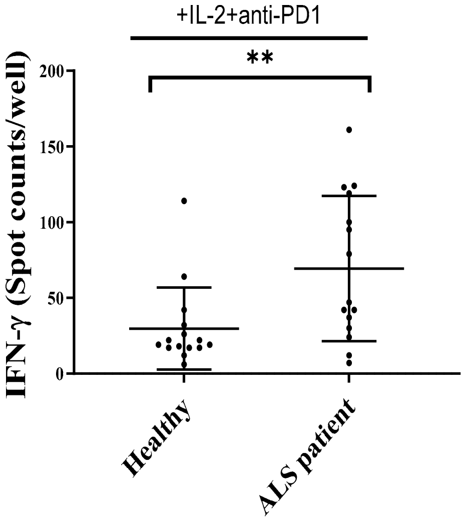

+IL-2+anti-PD1
**
IFN-γ (Spot counts/well)
200
150
100
50
0
Healthy
ALS patient

Figure S3

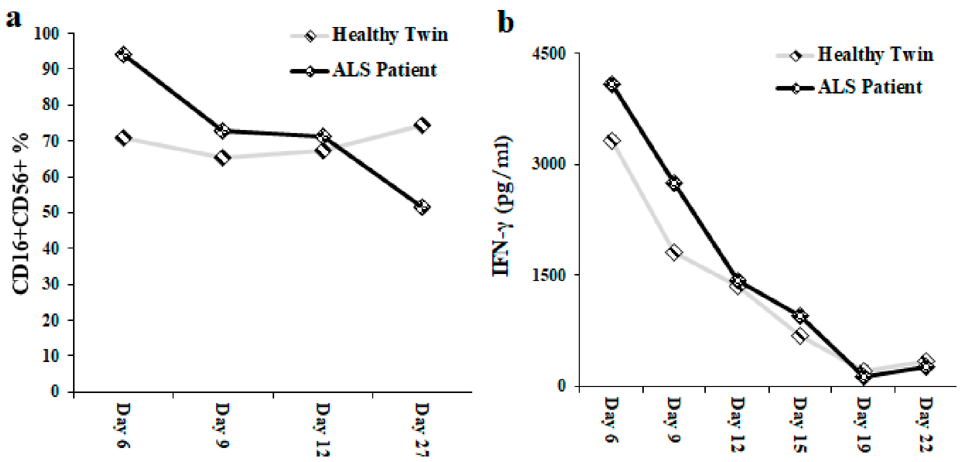
a
CD16+CD56+ %
100
90
80
70
60
50
40
30
20
10
0
Healthy Twin
ALS Patient
Day 6
Day 9
Day 12
Day 27
b
IFN-γ (pg/ml)
4500
3000
1500
0
Healthy Twin
ALS Patient
Day 6
Day 9
Day 12
Day 15
Day 19
Day 22

Figure S4

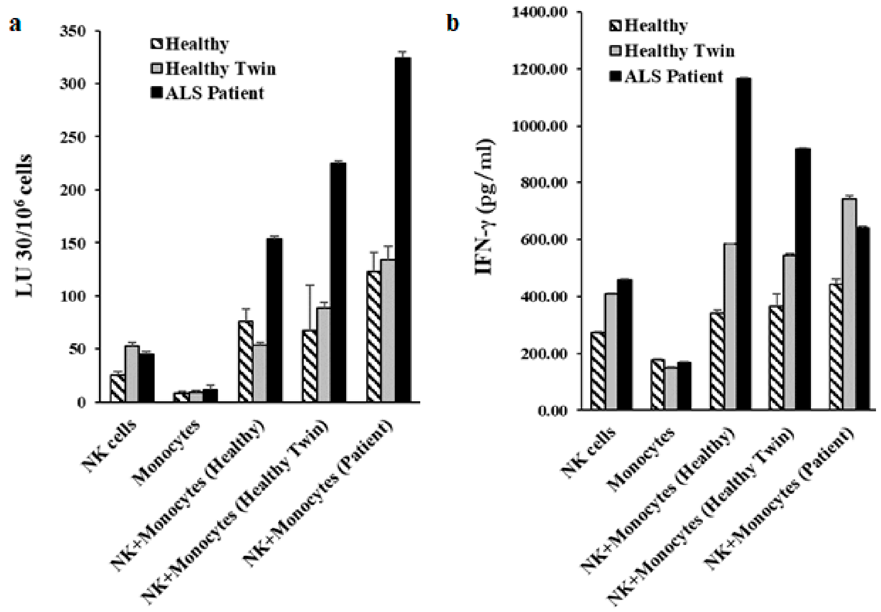
a
350
300
250
200
150
100
50
0
LU 30/10⁶ cells
Healthy
Healthy Twin
ALS Patient
NK cells
Monocytes
NK+Monocytes (Healthy)
NK+Monocytes (Healthy Twin)
NK+Monocytes (Patient)
b
1400.00
1200.00
1000.00
800.00
600.00
400.00
200.00
0.00
IFN-γ (pg/ml)
Healthy
Healthy Twin
ALS Patient
NK cells
Monocytes
NK+Monocytes (Healthy)
NK+Monocytes (Healthy Twin)
NK+Monocytes (Patient)

Figure S5

a

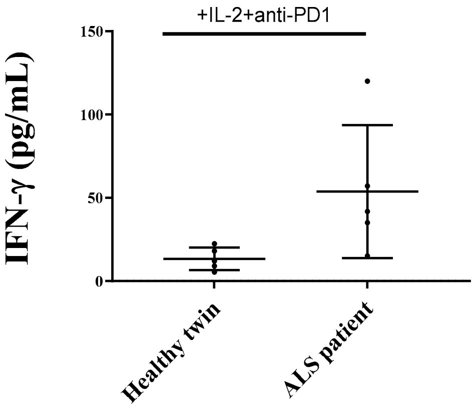

b

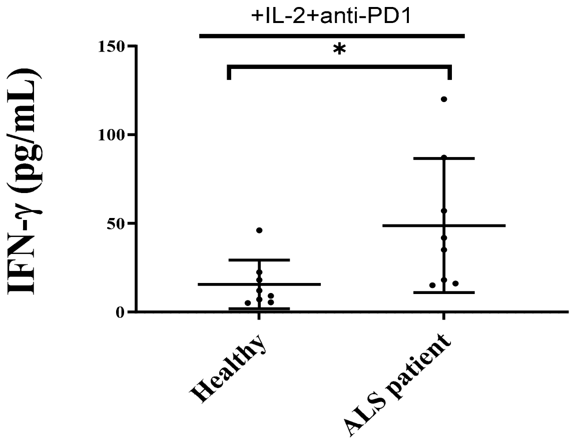

c

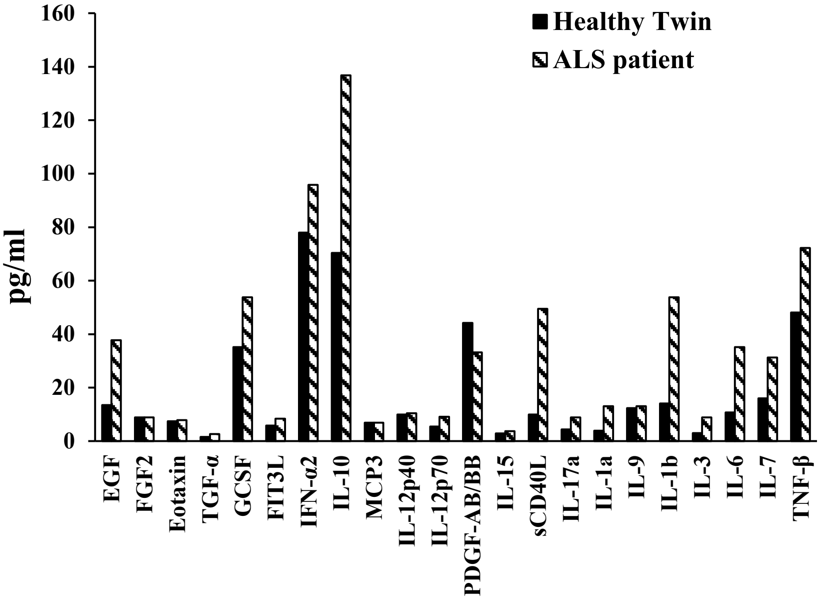

d

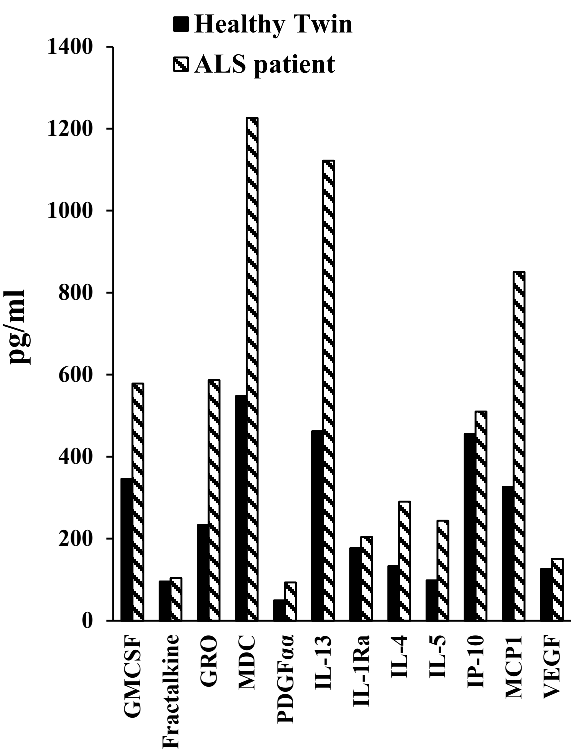

Healthy Twin
ALS patient
pg/ml
1400
1200
1000
800
600
400
200
0
GMCSF
Fractalkine
GRO
MDC
PDGFαα
IL-13
IL-1Ra
IL-4
IL-5
IP-10
MCP1
VEGF

e

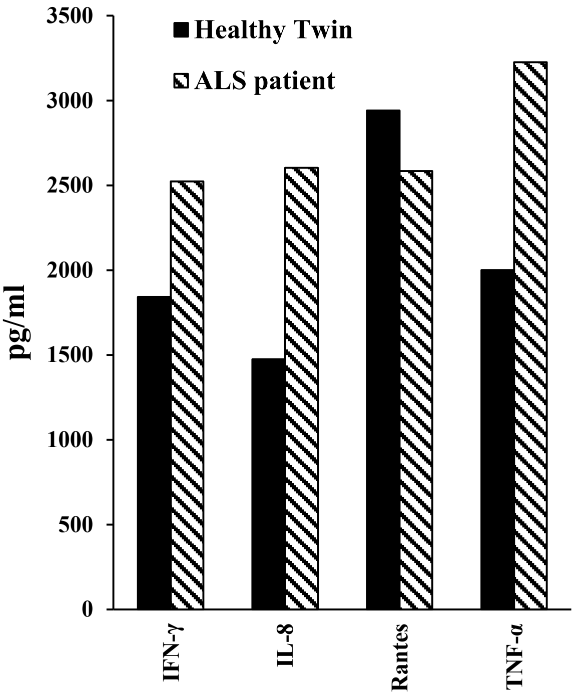

Healthy Twin
ALS patient
pg/ml
3500
3000
2500
2000
1500
1000
500
0
IFN-γ
IL-8
Rantes
TNF-α

Figure S6

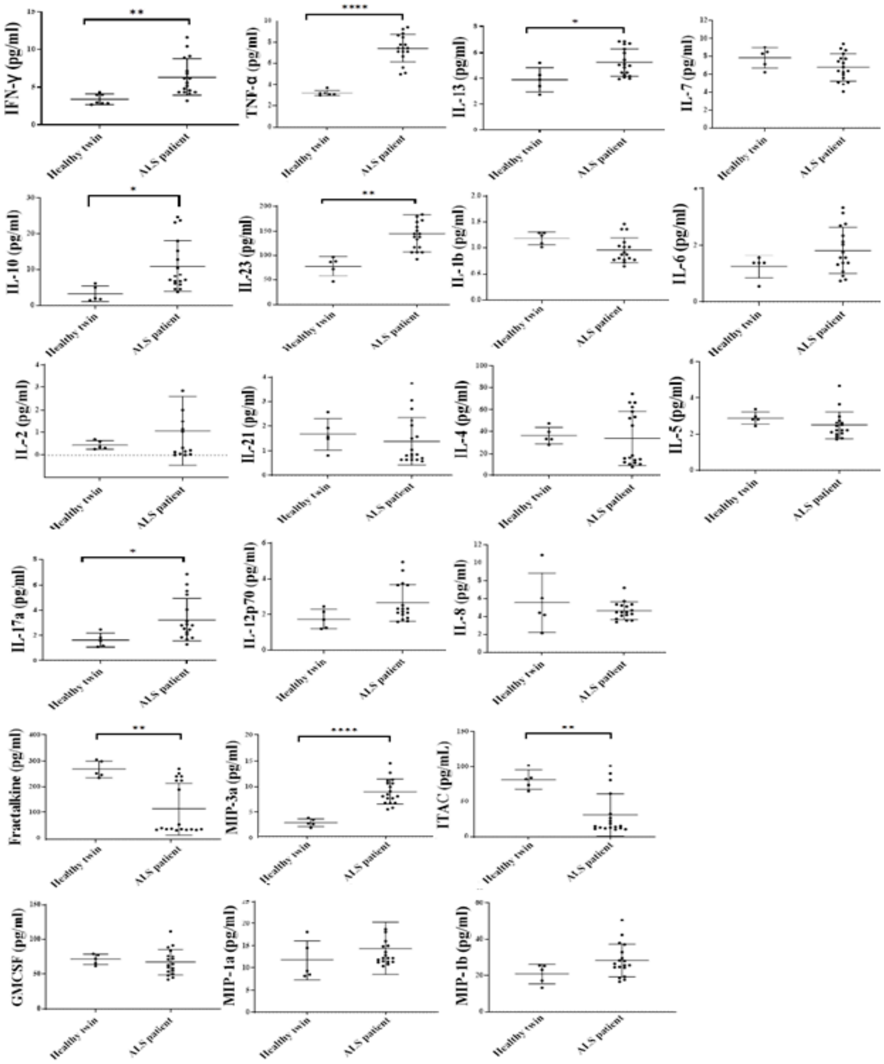
IFN-γ (pg/ml)
TNF-α (pg/ml)
IL-13 (pg/ml)
IL-7 (pg/ml)
IL-10 (pg/ml)
IL-23 (pg/ml)
IL-1b (pg/ml)
IL-6 (pg/ml)
IL-2 (pg/ml)
IL-21 (pg/ml)
IL-4 (pg/ml)
IL-5 (pg/ml)
IL-17a (pg/ml)
IL-12p70 (pg/ml)
IL-8 (pg/ml)
Fractalkine (pg/ml)
MIP-3a (pg/ml)
ITAC (pg/mL)
GMCSF (pg/ml)
MIP-1a (pg/ml)
MIP-1b (pg/ml)
Healthy twin
ALS patient

Figure S7

a

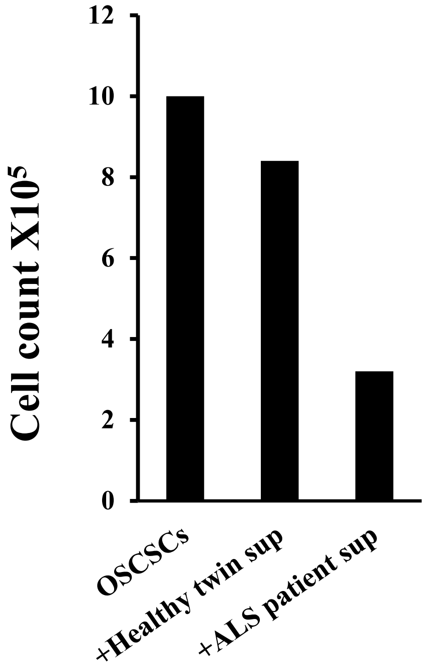

b

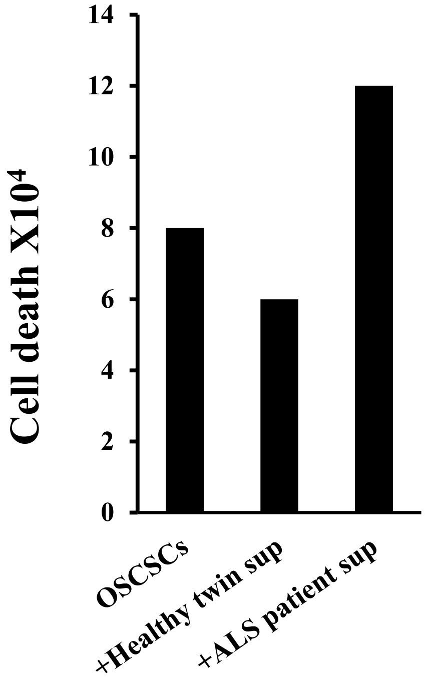

c

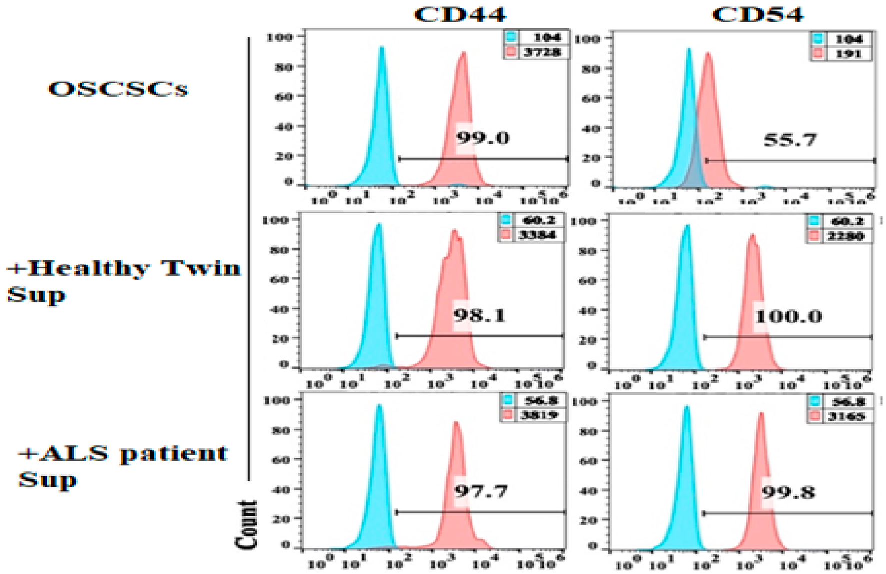

Figure S8

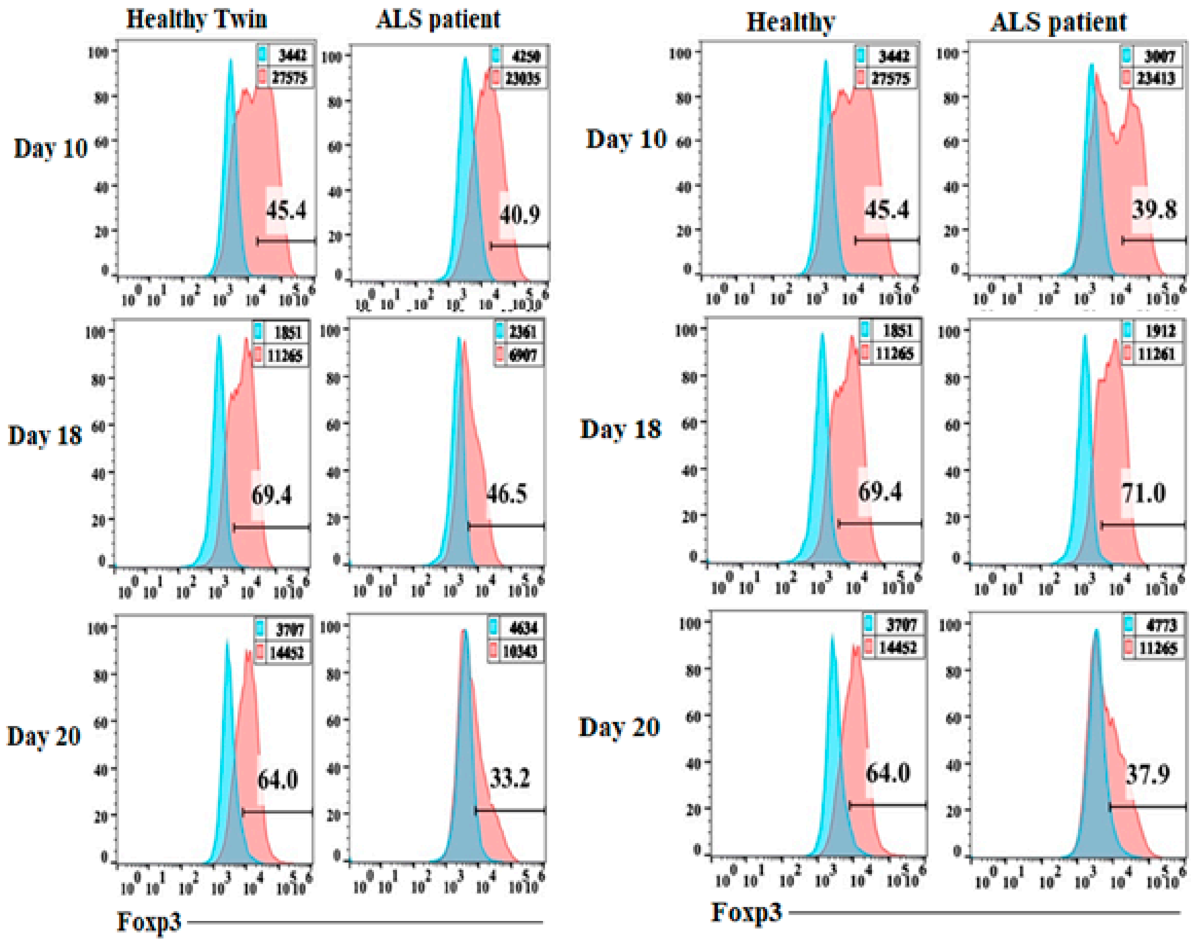
Healthy Twin
ALS patient
Healthy
ALS patient
Day 10
Day 18
Day 20
Day 10
Day 18
Day 20
3442
27575
45.4
4250
23035
40.9
3442
27575
45.4
3007
23413
39.8
1851
11265
69.4
2361
6907
46.5
1851
11265
69.4
1912
11261
71.0
3707
14452
64.0
4634
10343
33.2
3707
14452
64.0
4773
11265
37.9
Foxp3
Foxp3

Figure S9

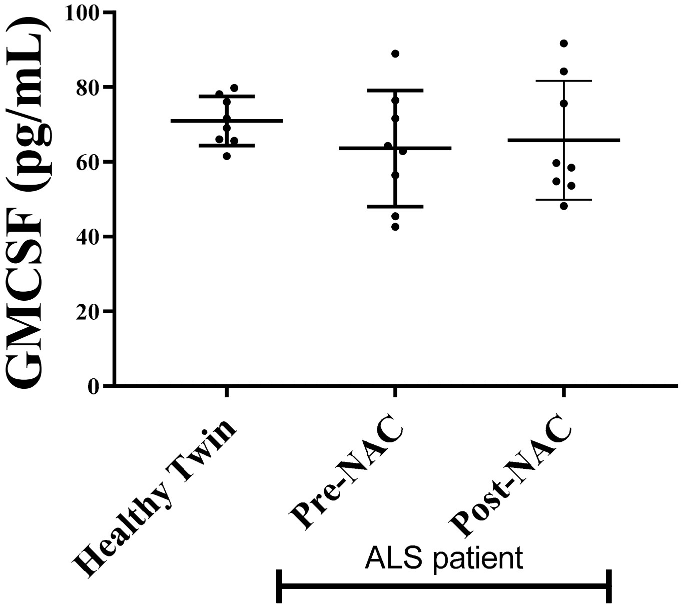
GMCSF (pg/mL)
100
80
60
40
20
0
Healthy Twin
Pre-NAC
Post-NAC
ALS patient
